# Supplementary material for: Drosophila TMEM63 and mouse TMEM63A are lysosomal mechanosensory ion channels
Source: Nat Cell Biol. 2024 Feb 22;26(3):393–403. doi: 10.1038/s41556-024-01353-7 (PMC10940159; doi:10.1038/s41556-024-01353-7)
Supplement: Supplementary file 1 — Reporting Summary [file 41556_2024_1353_MOESM1_ESM.pdf]

nature portfolio

Corresponding author(s): Yuh Nung Jan

Last updated by author(s): Dec 26, 2023

Reporting Summary

Nature Portfolio wishes to improve the reproducibility of the work that we publish. This form provides structure for consistency and transparency in reporting. For further information on Nature Portfolio policies, see our Editorial Policies and the Editorial Policy Checklist.

Statistics

For all statistical analyses, confirm that the following items are present in the figure legend, table legend, main text, or Methods section.

n/a	Confirmed	
☐	☒	The exact sample size (*n*) for each experimental group/condition, given as a discrete number and unit of measurement
☐	☒	A statement on whether measurements were taken from distinct samples or whether the same sample was measured repeatedly
☐	☒	The statistical test(s) used AND whether they are one- or two-sided<br>*Only common tests should be described solely by name; describe more complex techniques in the Methods section.*
☐	☒	A description of all covariates tested
☐	☒	A description of any assumptions or corrections, such as tests of normality and adjustment for multiple comparisons
☐	☒	A full description of the statistical parameters including central tendency (e.g. means) or other basic estimates (e.g. regression coefficient) AND variation (e.g. standard deviation) or associated estimates of uncertainty (e.g. confidence intervals)
☐	☒	For null hypothesis testing, the test statistic (e.g. *F*, *t*, *r*) with confidence intervals, effect sizes, degrees of freedom and *P* value noted<br>*Give P values as exact values whenever suitable.*
☒	☐	For Bayesian analysis, information on the choice of priors and Markov chain Monte Carlo settings
☒	☐	For hierarchical and complex designs, identification of the appropriate level for tests and full reporting of outcomes
☐	☒	Estimates of effect sizes (e.g. Cohen's *d*, Pearson's *r*), indicating how they were calculated

Our web collection on statistics for biologists contains articles on many of the points above.

Software and code

Policy information about availability of computer code

Data collection	Confocal images were taken by the Olympus FV3000 with the FV31S-SW (version 2.6) software or Leica SP8 with the LAS X (version 4.1.1) software. Calcium imaging data were taken under ORCA-Flash4.0 Hamamatsu digital camera (version C13440) paired with Olympus IX73 microscope with Micro-manager (version 2.0.0) software or Olympus SpinSR spinning disk con-focal microscope with the cellSens (version 4.2) software. Electrophysiological recordings were performed with an Axopatch 700B amplifier and a Digidata 1440A or Digidata 1550B digitizer (Molecular Devices). Electrophysiology data were acquired by pClamp and Clampfit (Molecular Devices, version 10.4) softwares.
Data analysis	Origin 2021 was utilized for statistical analysis and generating graphs. Image J Fiji 2.1.0 was used for image quantification. For co-localization quantification, Pearson's correlation coefficient was quantified by Coloc2 plugin in ImageJ. Phylogenetic tree was constructed by the Phylogeny tool at EMBL-EBI. The analysis of correlation between lysosomal curvature and calcium intensity was performed in Matlab (version R2020a) with codes available at Github (https://github.com/weichensteven/lysosome_curvature_calcium_correlation_analysis).

For manuscripts utilizing custom algorithms or software that are central to the research but not yet described in published literature, software must be made available to editors and reviewers. We strongly encourage code deposition in a community repository (e.g. GitHub). See the Nature Portfolio guidelines for submitting code & software for further information.

Data

Policy information about availability of data

All manuscripts must include a data availability statement. This statement should provide the following information, where applicable:

- Accession codes, unique identifiers, or web links for publicly available datasets
- A description of any restrictions on data availability
- For clinical datasets or third party data, please ensure that the statement adheres to our policy

All data of this study are available in the main text or the extended materials with with numeric data of graphs and uncropped scans of all blots and gels shown in Source Data files. The genotypes of the animals used in figures and the sequences of oligonucleotides used in Methods are listed in Supplementary Table 1 and 2, respectively. For Extended Data Fig. 2b, datasets analyzed are available in the Fly Cell Atlas repository with the following weblink https://scope.aertslab.org/#/FlyCellAtlas/*/welcome. The two papers related to the dataset were cited in the Methods references of the manuscript.

Human research participants

Policy information about studies involving human research participants and Sex and Gender in Research.

Reporting on sex and gender	N/A
Population characteristics	N/A
Recruitment	N/A
Ethics oversight	N/A

Note that full information on the approval of the study protocol must also be provided in the manuscript.

Field-specific reporting

Please select the one below that is the best fit for your research. If you are not sure, read the appropriate sections before making your selection.

☒ Life sciences ☐ Behavioural & social sciences ☐ Ecological, evolutionary & environmental sciences

For a reference copy of the document with all sections, see nature.com/documents/nr-reporting-summary-flat.pdf

Life sciences study design

All studies must disclose on these points even when the disclosure is negative.

Sample size	No statistical method was used to predetermine sample size. Sample sizes were based on current standard in the field and previous studies (PMID: 23222543, PMID: 25959678, PMID: 30382938, PMID: 35780140). The sample sizes used in this paper are indicated in the corresponding figures or figure legends.
Data exclusions	The total number of the tested flies was counted after climbing assays, if there are dead flies after the experiments, the dead flies will not be counted.
Replication	All experiments were performed at least three times. All attempts at replication were successful.
Randomization	Animals or cells were allocated based on genotypes or treatments, which were described in the corresponding figure legends. Images were obtained randomly and were taken from random regions of prepared samples.
Blinding	The investigators who collected and analyzed the image data were blinded to group allocation. The investigators were blinded to the genotypes during the behavioural assays. Since only one single investigator typically performed all procedures of the electrophysiological experiments, the investigator was not blinded to the group allocation in electrophysiology.

Reporting for specific materials, systems and methods

We require information from authors about some types of materials, experimental systems and methods used in many studies. Here, indicate whether each material, system or method listed is relevant to your study. If you are not sure if a list item applies to your research, read the appropriate section before selecting a response.

Materials & experimental systems

n/a	Involved in the study
☐	☒ Antibodies
☐	☒ Eukaryotic cell lines
☒	☐ Palaeontology and archaeology
☐	☒ Animals and other organisms
☒	☐ Clinical data
☒	☐ Dual use research of concern

Methods

n/a	Involved in the study
☒	☐ ChIP-seq
☒	☐ Flow cytometry
☒	☐ MRI-based neuroimaging

Antibodies

Antibodies used

The primary antibodies used in the immunostaining experiments were: Mouse anti-GFP antibody (Roche, # 11814460001); Chicken anti-mCherry antibody (Novus Biologicals, # NBP2-25158); Mouse anti-BRP antibody (Developmental Studies Hybridoma Bank, # nc82). The secondary antibodies for immunostaining were anti-mouse labeled by Alexa 488 (ThermoFisher # A28175), or Cy3 (Jackson ImmunoResearch Labs # 115-165-146) and anti-chicken labeled by Alexa 647 (Jackson ImmunoResearch Labs, # 103-605-155). The primary antibody used in the Western blots were: Mouse anti-GFP antibody (Roche, # 11814460001); Rabbit anti-mCherry antibody (Abcam, # ab167453); Rabbit anti-Ref2P antibody (Abcam, # ab178440); Rabbit anti-TMEM63A antibody (Novus Biologicals, # NBP2-57359); Mouse anti-GAPDH antibody (Proteintech, # 60004-1-Ig); Mouse anti-tubulin antibody (Sigma, # 9026). The secondary antibodies for the Western blots were anti-mouse HRP antibody (Jackson ImmunoResearch Labs, # 115-035-146); anti-rabbit HRP antibody (Jackson ImmunoResearch Labs, # 111-035-144).

Validation

(1) Mouse anti-GFP antibody (Roche, # 11814460001; WB: 1:2000; IF: 1:500; https://www.sigmaaldrich.com/US/en/product/roche/11814460001#product-documentation)
(2) Chicken anti-mCherry antibody (Novus Biologicals, # NBP2-25158; IF: 1:300; https://www.novusbio.com/products/mcherry-antibody_nbp2-25158).
(3) Rabbit anti-mCherry antibody (Abcam, # ab167453; WB: 1:2000; https://www.abcam.com/products/primary-antibodies/mcherry-antibody-ab167453.html);
(4) Mouse anti-BRP antibody (1:100, Developmental Studies Hybridoma Bank, # nc82; IF: 1:100; https://dshb.biology.uiowa.edu/nc82).
(5) Rabbit anti-Ref2P antibody (Abcam, # ab178440; WB: 1:500; https://www.abcam.com/products/primary-antibodies/ref2p-antibody-ab178440.html).
(6) Mouse anti-GAPDH antibody (Proteintech, # 60004-1-Ig; WB: 1:2000; https://www.ptglab.com/products/GAPDH-Antibody-60004-1-Ig.htm);
(7) Mouse anti-tubulin antibody (Sigma, # 9026; WB: 1:2000; https://www.sigmaaldrich.com/US/en/product/sigma/t9026).
(8) Rabbit anti-TMEM63A antibody (Novus Biologicals, # NBP2-57359; WB: 1:200; https://www.novusbio.com/products/tmem63a-antibody_nbp2-57359). This antibody was also validated by experiments in this paper, because TMEM63A proteins could be detected through this antibody in N2a WT cells expressing endogenous TMEM63A (Fig. 6b) but not detected in the MsTmem63-KO cells (Extended Data Fig. 9f).

Eukaryotic cell lines

Policy information about cell lines and Sex and Gender in Research

Cell line source(s)

The following cell lines were from ATCC: S2 cells (ATCC, # CRL-1963), HEK293 cells (ATCC, # CRL-1573), HEK293T cells (ATCC, # CRL-3216), and SH-SY5Y cells (ATCC, # CRL-2266). BV-2 cells were obtained from Dr. Li Gan lab and originally from InterLab Cell Line Collection, Banca Biologica e Cell Factory (ICLC, # ATL03001). N2a cells were obtained from Dr. Nevan Krogan lab and originally from ATCC (ATCC, # CCL-131).

Authentication

Cell line authentication was performed by the supplier through STR. In addition, S2 cells, HEK293 cells and N2a cells have distinct morphology and growing rate, which was used to distinguish the cell types during cell maintenance.

Mycoplasma contamination

Cell lines were negative for mycoplasma through PCR-based mycoplasma detection.

Commonly misidentified lines
(See ICLAC register)

No commonly misidentified cell lines were used.

Animals and other research organisms

Policy information about studies involving animals; ARRIVE guidelines recommended for reporting animal research, and Sex and Gender in Research

Laboratory animals

(1) Fruit flies (Drosophila melanogaster) was used as the laboratory animal. Larvae or adults were used with the developmental stages or ages indicated in figures or figure legends.
(2) The following fly strains were obtained from the Bloomington Stock Center: stock # 39668 (UAS-spin.myc-GFP), stock # 7011 (Cg-GAL4), stock # 48183 (GMR51B08-GAL4), stock # 32194 (UAS-mCD8-GFP) and stock # 58772 (UAS-Piezo-GFP).
(3) The following stocks were obtained from other labs: UAS-GFP-LAMP1, Cg-GAL4 UAS-GFP-LAMP1.
(4) The following transgenic flies were generated in this study by P-element-mediated germline transformation or phiC31-mediated

integration: Tmem63-Gal4, UAS-Tmem63-GFP, UAS-Tmem63.
(5) The Tmem63 mutant flies were generated through ends-out homologous recombination or CRISPR/Cas9 technology.
(6) The DmTmem63-GFP and DmTmem63-mCherry knock-in flies were generated via CRISPR/Cas9 system.

Wild animals	This study did not involve wild animals.
Reporting on sex	none
Field-collected samples	none
Ethics oversight	No ethical approval was required for the fly work.

Note that full information on the approval of the study protocol must also be provided in the manuscript.
