# Supplementary figures and images for: Drosophila TMEM63 and mouse TMEM63A are lysosomal mechanosensory ion channels
Source: Nat Cell Biol. 2024 Feb 22;26(3):393–403. doi: 10.1038/s41556-024-01353-7 (PMC10940159; doi:10.1038/s41556-024-01353-7)

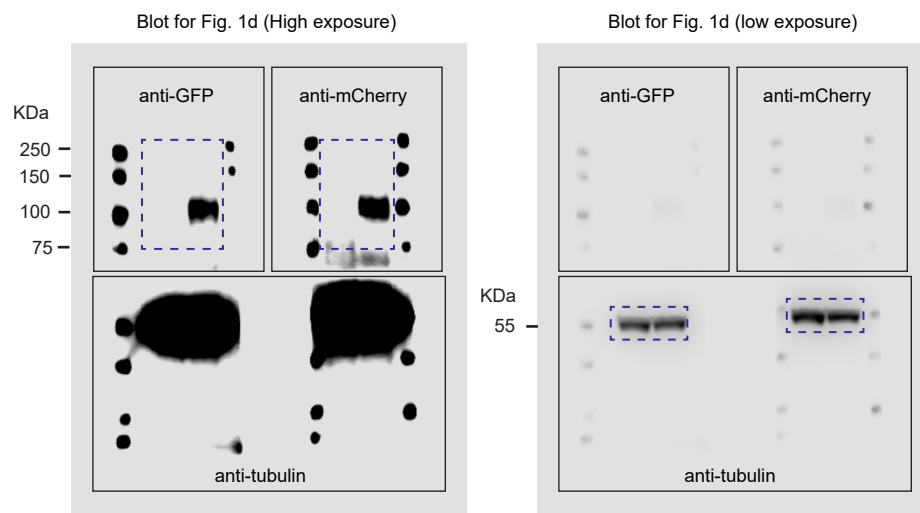

The dotted blue boxes indicate the regions shown in the figures.

Supplement: Supplementary file 5 — Unprocessed western blots. [file 41556_2024_1353_MOESM5_ESM.pdf]

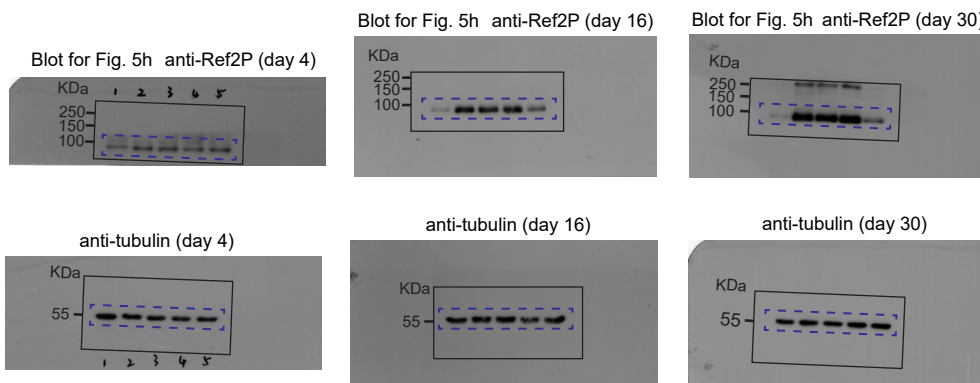

The dotted blue boxes indicate the regions shown in the figures.

Supplement: Supplementary file 10 — Unprocessed western blots. [file 41556_2024_1353_MOESM10_ESM.pdf]

Blot for Fig. 6b

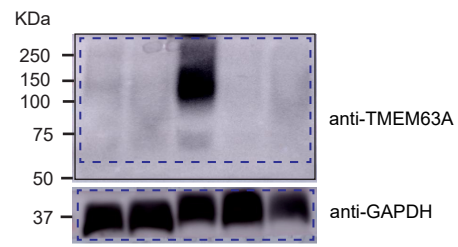

The dotted blue boxes indicate the regions shown in the figures.

Supplement: Supplementary file 12 — Unprocessed western blots. [file 41556_2024_1353_MOESM12_ESM.pdf]

Blot for Extended Data Fig. 9f

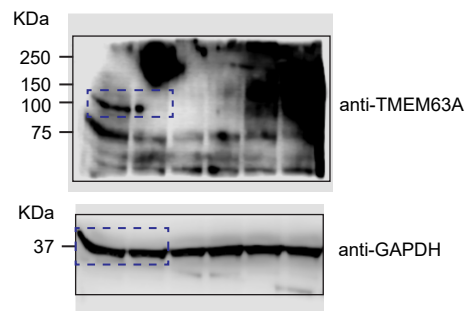

The dotted blue boxes indicate the regions shown in the figures.

Supplement: Supplementary file 20 — Unprocessed western blots. [file 41556_2024_1353_MOESM20_ESM.pdf]
