# Supplementary material for: Drosophila TMEM63 and mouse TMEM63A are lysosomal mechanosensory ion channels
Source: Nat Cell Biol. 2024 Feb 22;26(3):393–403. doi: 10.1038/s41556-024-01353-7 (PMC10940159; doi:10.1038/s41556-024-01353-7)
Supplement: Supplementary file 15 — Unprocessed gels. [file 41556_2024_1353_MOESM15_ESM.pdf]

Gel for Extended Data Fig. 4b

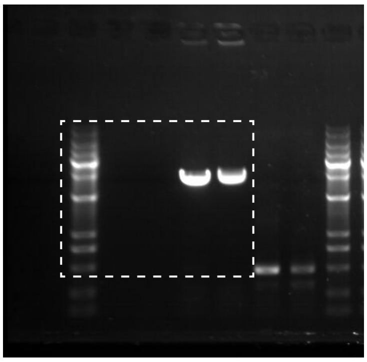

Gel for Extended Data Fig. 4d

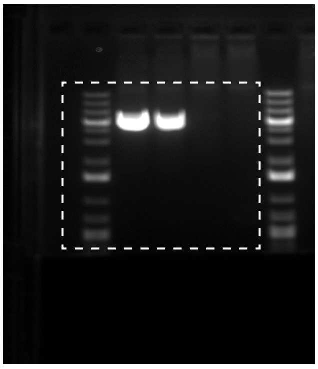

The dotted white boxes indicate the regions shown in the figures.
